# MRI for PIP implant rupture: appearances and rupture rate

**DOI:** 10.1186/bcr3297

**Published:** 2012-11-09

**Authors:** V Helyar, S McWilliams, C Burke, G Charles-Edwards

**Affiliations:** 1 Guy's and St Thomas' NHS Foundation Trust, London, UK

## Introduction

The Poly Implant Prosthèse breast implants contain an unlicensed silicone and are associated with elevated rupture rate. We examined the performance of our MR breast protocol for implant assessment and quantified radiological rupture rate.

## Methods

This was a retrospective review of 145 patients receiving an implant protocol MR breast between January and April 2012 at Guy's Hospital. Scans were reported by two consultant radiologists. Patient symptoms, MR findings and origin of surgery were recorded. Data were analysed with descriptive statistics.

## Results

Twenty-one per cent (30/145) showed unilateral rupture, 46% (14/30) bilateral rupture. Of those with a rupture, only four were symptomatic. Seventy-seven per cent (23/30) of ruptures were intra-capsular, 67% (20/30) showed evidence of silicone adenitis. MR findings show complete agreement with the explantation data gathered to date.

## Conclusion

These preliminary data support the use of our MR protocol for the assessment of breast implant integrity.

